# Chronic pain has a small influence and mood has no influence on vibrotactile perception thresholds among working women

**DOI:** 10.1002/mus.21697

**Published:** 2010-06-17

**Authors:** Helena Sandén, B Gunnar Wallin, Mats Hagberg

**Affiliations:** 1 Occupational and Environmental Medicine, Sahlgrenska School of Public Health and Community Medicine, University of GothenburgBox 414, SE 405 30 Göteborg, Sweden; 2 Institute of Neuroscience and Physiology, Sahlgrenska Academy, University of GothenburgSweden

**Keywords:** chronic pain, upper limb, vibrotactile perception threshold, mood, nerve conduction

## Abstract

In chronic diffuse upper limb pain physical abnormalities are usually absent. The aims of our study were to investigate: (1) the function of somatosensory pathways and (2) the influence of mood on vibration perception. Measurements were made of: (i) vibrotactile perception thresholds (VPTs) and nerve conduction in working women with (*n* = 35) and without (*n* = 65) chronic diffuse upper limb pain, and (ii) perceived stress and energy using a two-dimensional mood adjective checklist. The groups did not differ in any nerve conduction measurements. Women with chronic pain had raised VPTs in the radial and ulnar nerve areas, but not in the median nerve area. Neither perceived stress nor energy appeared to influence the VPT. Increases of VPTs in chronic diffuse upper limb pain may be due to peripheral nerve affliction, but our findings support the idea that they may also be secondary to pain and may be related to a central nervous mechanism. Muscle Nerve, 2010

Although chronic diffuse upper limb pain is common, its etiology is sometimes uncertain. The symptoms that are sometimes considered to be work-related include varying degrees of pain, weakness, and numbness/tingling. Chronic diffuse upper limb pain may be due to minor nerve entrapments, but a standard physical examination is unlikely to demonstrate abnormality.

Patients and computer users with nonspecific arm pain have been reported to have raised vibrotactile perception thresholds (VPTs) in the hand within the areas of the median and ulnar nerves.^1^–^3^ In addition, office workers who experience neck pain have also recently been reported to have raised VPTs in the hand within the area of the radial nerve.^4^ Decreased vibration sensitivity can be an early sign of peripheral neuropathy, but VPTs depend on the integrity of the entire somatosensory pathway (peripheral mechanoreceptors, peripheral large myelinated sensory nerves, central nervous system, CNS^5^). Laursen et al.^6^ and Tucker et al.^7^ found raised VPTs in the contralateral limb in patients with upper limb disorders, indicating alterations in the CNS. The vibration threshold test is psychophysical in nature, since it has an objective physical stimulus but a subjective response from the tested subject. Thus, in contrast to nerve conduction measurements (testing peripheral large myelinated nerve fibers), the vibration threshold test requires cooperation from the subject and is affected by attention, concentration, and motivation. Changes in VPTs may therefore be due to altered mood. A literature search in PubMed revealed no publication on this issue. To our knowledge, these factors have not been studied previously.

The aim of this study was to investigate the function of the somatosensory pathways using vibration threshold testing and nerve conduction measurements in the upper extremity in working women with and without chronic diffuse upper limb pain. We were also interested in examining whether mood influences the result of vibration threshold testing, and so prior to the vibration threshold test, perceived stress and energy were assessed using a two-dimensional mood adjective checklist.

## SUBJECTS AND METHODS

### Subjects

The study was approved by the human ethics committee at the University of Gothenburg. Female subjects were invited to participate in the investigation by means of advertisements posted on personnel notice boards. The invitation referred to working women with and without chronic upper limb pain. The rationale for choosing women was that they are at a greater risk for chronic upper limb pain.^8^–^10^ The subjects worked as secretaries or nurses in different healthcare facilities in the southwest of Sweden and were also part of a study on computer keyboard work.^11^ The inclusion criteria were thus: female gender, working as a secretary or nurse, with or without chronic pain in upper limb of the dominant hand. The exclusion criteria were disease or medical treatment with possible effect on peripheral nerves.

A total of 127 female participants entered the study. In all, 51 had chronic pain for more than 3 months, and 76 were normally pain-free. Six participants had pain elsewhere in the body such as lower back, leg, knee, and the nondominant arm/hand, and these six were excluded. Five were excluded because of disorders predisposing to upper-limb conditions and nerve affliction (multiple sclerosis, diabetes, rheumatoid arthritis, non-Hodgkin's lymphoma, vitamin B_12_ deficiency). Five subjects with symptoms of carpal tunnel syndrome were excluded after the nerve conduction measurements [sensory latency from palm to wrist (third finger stimulation) greater than 1.73 ms at a distance of 60 mm]. One subject was excluded, as she was diagnosed with polyneuropathy after the nerve conduction test. Ten were excluded from the analysis because of missing data. The final study population thus included 35 individuals with chronic diffuse upper limb pain, age 30–65 (median 46) years and 65 individuals without chronic pain, age 24–57 (median 42) years. The medications being taken by the participants with chronic pain included nonnarcotic analgesics (2), antidepressants (1), antihistamines (1), bronchodilators (1), thyroid hormones (1), diuretics (1), and hypolipidemic agents (2). Medications being taken by the controls included antidepressants (1), hormonal contraceptives (6), thyroid hormones (2), angiotensin receptor blockers (1), and sumatriptan (1). Hence, two participants were on long-term treatment with antidepressants due to recurring depression; at the time of the examination there were no clinical signs of ongoing depression.

### Methods

**FIGURE 1:**
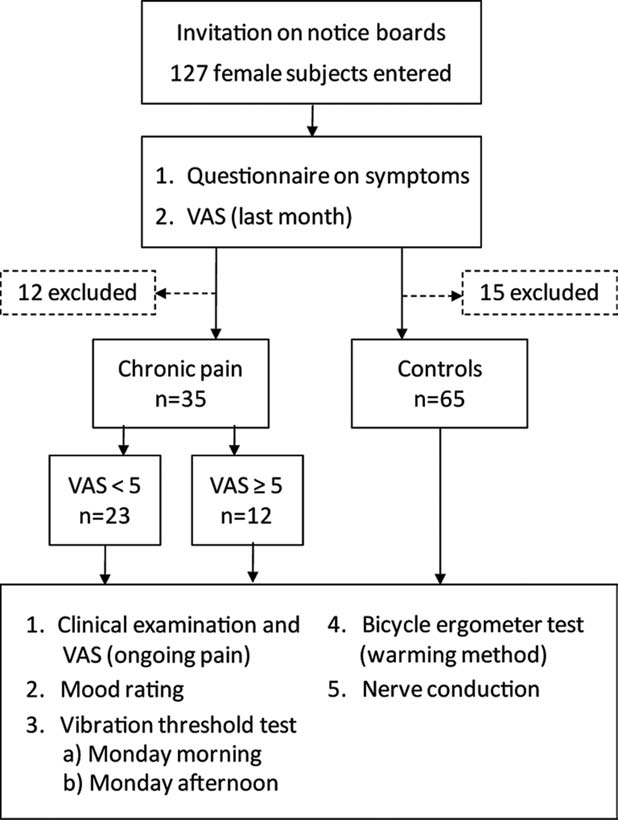
Flow chart.

A flow chart of the study is presented in Figure 1. Each participant completed a questionnaire on symptoms in the upper extremity and chronic pain for more than 3 months. Average pain intensity during the last month was measured using a 10 cm visual analog scale (VAS) and the subjects with chronic upper limb pain were then divided into two subgroups with a cutpoint between mild and moderate/severe pain.^12^ The subjects underwent a brief clinical examination by a physician after and in most cases on the same day as the vibration threshold testing. It was occasionally not possible to perform the clinical examination on the same day, and in these cases the medical examination took place within the same week. In connection with the medical examination, ongoing pain intensity was measured using the VAS.

### Physical Examination

The physical examination of neck and upper limbs (shoulder, elbow, wrist, hand, finger) included the following steps: (1) inspection; (2) testing for range of active and passive motion; (3) testing for muscle contraction, pain, and muscle strength; (4) palpation of muscle tendons, insertions, and joints; (5) bedside neurologic examination, including muscle stretch reflexes (biceps, triceps, brachioradialis, achilles), sensory exam in hand/fingers evaluating different kinds of sensation, including light touch (cotton wool), two-point-discrimination and temperature (a tuning fork at room temperature should be perceived as cold on the digital pulp of index and fifth fingers); (6) specific tests; Spurling test (neck compression test), cervical spine Laségue test (extending the plexus by axial compression of the acromion with simultaneous lateral flexion of the subjects's cervical spine towards the contralateral side), Roos' test (abduction external rotation test), bursa test for shoulder bursitis, pronator compression test, palpation at the arcade of Frohse, Maudsley's test (middle finger extension test), Finkelstein's test, Phalen's test (wrist compression test), and Tinel's test. A detailed list of the physical examination and details concerning tests are available at our homepage (http://www.amm.se/vptstudy). Further details concerning some of these tests can also be found in Nilsson.^13^

### Mood Ratings

Perceived stress and energy were assessed using a two-dimensional mood adjective checklist, the Stress-Energy (SE) Questionnaire.^14^,^15^ This questionnaire has been used in several Swedish studies of occupational stress.^16^–^19^ It is an adjective checklist that is designed to be used for describing mood during work. This checklist was used just before the vibration testing on a Monday morning. Twelve adjectives represent two fundamental dimensions; stress and energy. The overall question to be answered by the checklist was: “How did you feel over the last 10 minutes?” The participants indicated on a six point scale (0–5) how well each adjective described their state. The stress dimension used the following adjectives: “tense,” “stressed,” “pressured,” “relaxed,” “rested,” and “calm” while the energy dimension used “focused,” “energetic,” “active,” “inefficient,” “dull,” and “passive.” Before analysis the scores for the negative items (inefficient, dull, passive, relaxed, rested, and calm) were reversed, with a score of 5 being mapped to a score 0, a score of 4 to a score of 1, and so on. Stress and energy scores were calculated as mean ratings of the six items in each dimension after reversal of the negative items. Cronbach's alpha for stress was 0.85 and for energy 0.69. The neutral points of the scales have previously been calculated; the neutral point for the stress scale (neither stressed nor calm) is 2.4 and the neutral point for the energy scale is 2.7.^15^

### Vibration Threshold Test

A handheld vibrometer (type IV, Somedic AB, Stockholm, Sweden), operating at a frequency of 120 Hz and a tissue displacement range of 0.1–400 μm, was used to deliver mechanical stimulation to the hand. The vibrating probe was 1 cm in diameter, and the amplitude of the vibration was displayed digitally. The validity and reliability of the vibration threshold test have been established in previous studies.^20^–^22^ Readings were taken at five sites on the dominant hand: (1) the distal pad of the index finger (median nerve); (2) the distal pad of the 5th finger (ulnar nerve); (3) the dorsum of the 5th metacarpal bone (ulnar nerve); (4) the dorsum of the 2nd metacarpal bone (radial nerve); and (5) the palmar aspect between the 1st and 2nd metacarpal bones (median nerve). During the measurements at the metacarpal bones and at the palmar aspect between the 1st and 2nd metacarpal bones the probe was placed perpendicular to the skin surface, and a pressure display enabled the applied pressure to be standardized to ≈8 N/cm^2^. During the measurements at the fingertips the subject was asked to place the distal pad of the test finger over the probe and push down with a force of 0.4 N, visually controlled by the pressure display, which had been calibrated with a weight of 41 g.

All vibration threshold examinations were performed by one assistant who was blinded both to the group of the subjects and to the results of the preceding examination. The subjects were seated comfortably and examined in a quiet room without distractions. They could not see the vibrometer display. The stimulus was increased at a constant rate until the subject could just detect vibration. From this threshold the stimulus was then decreased until the subject could no longer feel the vibration. This ramping up and down was repeated four times. The means of four readings for both detection and loss of vibration stimulation at each site were calculated, and the average of the two figures was taken as a measure of VPT.^23^

The subjects were tested both on a Monday morning after a weekend off work and on a Monday afternoon after at least 4 hours of working with either a computer keyboard or in their usual duties of nursing.

The power of the study to detect a difference of 0.3 μm (*P* < 0.05) within the area of the median nerve was 99%, the corresponding power to detect a difference of 0.15 μm was 61%. The power to detect a difference of 0.15 μm within the area of the ulnar and radial nerves was >99.9%.

### Nerve Conduction Test

In order to ensure an adequate hand temperature and minimize temperature as a source of error,^24^,^25^ nerve conduction testing was preceded by a bicycle ergometer test which has been shown to stabilize fingertip skin temperature at around 34°C.^26^ Skin temperature was measured at the tip of digit IV. During the nerve conduction test the subjects were covered by warm blankets.

Nerve conduction measurements were made on the dominant hand using an electromyography (EMG) apparatus (Keypoint Portable, Keypoint Software v. 3.0, Medtronic NeuroMuscular, Denmark). The test was performed by an experienced EMG technician who was blinded to the results of all other tests.

The median nerve motor conduction velocity was determined using surface electrodes for stimulation at the elbow and proximal to the wrist and for recording over the abductor pollicis brevis muscle. The distance between the recording and stimulation electrodes at the wrist was 7 cm. The F-wave latency was measured as the shortest latency obtained with 20 stimuli at the wrist. Sensory conduction velocity (SCV) of the median nerve was determined orthodromically from the second and third finger to the palm and the wrist, respectively, using surface electrodes mounted at fixed sites in a plastic splint held against the skin over the nerve. The ulnar SCV was measured from the fifth finger to the wrist using electrodes fixed in a similar splint as for the median nerve. Sural nerve SCV was also measured in order to control for nonsymptomatic polyneuropathy.

### Statistics

The *t*-test for comparison between independent groups was used in the analysis of the variables in the nerve conduction testing and mood ratings. The Wilcoxon rank sum test was used to compare the groups in the analysis of the VPTs, as the values were not normally distributed. In a multivariate analysis, linear regression of the VPTs was used to model the impact of individual exposure variables. Paired *t*-tests were used to compare each individual's first and second measurements. *P*-values <0.05 were considered significant. JMP 7® was used to perform all the analyses including power statistics.

## RESULTS

### Group Characteristics and Mood

The chronic diffuse upper limb pain group, which was significantly older than the control group (Table 1), passed the medical examination without signs of nervous disease. All subjects had nonspecific arm and/or neck pain without specific signs of disease (e.g., tenosynovitis, nerve entrapment, arthrosis). After analysis of the questionnaire regarding estimated average pain intensity during the last month (VAS), the chronic diffuse upper limb pain group—“chronic pain (all)”—was divided into two subgroups,^12^ “chronic pain (VAS ≥ 5)” (*n* = 12) and “chronic pain (VAS < 5)” (*n* = 23).

**Table 1:** Group characteristics: age, VAS (estimated average pain last month and ongoing pain in connection with medical examination), and mood

		Age (years)	VAS last month (cm)	VAS ongoing (cm)	Energy	Stress
Subject group	*n*	Median (range)	Mean (SD)	Mean (SD)	Mean (SD)	Mean (SD)
Controls	65	42 (24–57)	1.2 (1.5)	0.2 (0.7)	3.1 (0.7)	1.4 (0.7)
Chronic pain (VAS < 5)	23	48 (30–65)	2.4 (1.6)	2.5 (2.0)	3.2 (0.5)	1.4 (0.6)
Chronic pain (VAS ≥ 5)	12	42 (32–61)	6.1 (0.9)	2.8 (2.8)	3.2 (0.5)	2.1 (0.7)
Chronic pain (all)	35	46 (30–65)	3.7 (2.3)	2.6 (2.3)	3.2 (0.5)	1.6 (0.7)

The control group also passed the medical examination without signs of nervous system disease and had no history of pain with long duration in recent years, and only a few members of this group reported acute temporary pain in connection with medical examination and/or vibration threshold testing. When filling out the questionnaire, a few reported pain during the preceding month but in these cases the physician could not confirm any prolonged periods of pain when talking to the participant.

The VAS results regarding pain during the medical examination (on the same day as the vibration threshold testing) and average pain intensity during the last month are presented in Table 1. The chronic pain (VAS ≥ 5) group had significantly higher stress scores than the control group but the groups did not differ significantly in mean energy scores (Table 1).

### Vibration Threshold Test

**FIGURE 2:**
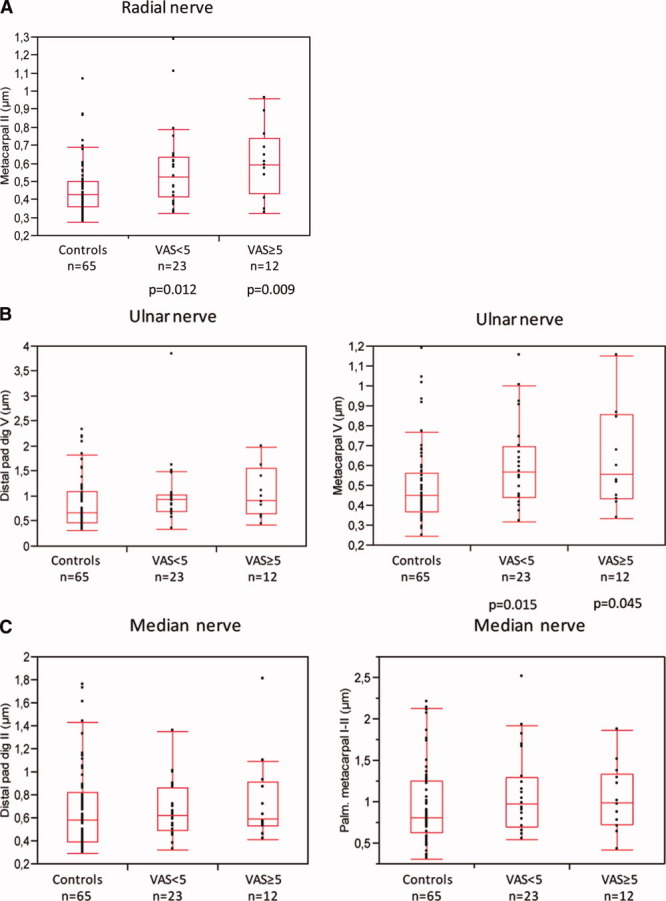
Vibrotactile perception thresholds in the area of the (**A**) radial, (**B**) ulnar, and (**C**) median nerve. Median values (–) are presented within the 25–75 interquartile box. The whiskers extend to the farthest point that is still within 1.5 interquartile ranges from the quartiles.

Compared to the control group, VPT was significantly higher among the chronic pain (all) group, the chronic pain (VAS < 5) group, and the chronic pain (VAS ≥ 5) group within the area of the radial nerve (*P*-values: 0.001, 0.012, 0.009, respectively) and within the area of the ulnar nerve at the metacarpal site (*P*-values: 0.004, 0.015, 0.045, respectively), but not at the finger tip (Fig. 2A–C). In the multivariate regression model analysis, with age, stress, and energy as covariates, the VPT was still significantly higher in the chronic pain (all) group (Table 2) and the chronic pain (VAS ≥ 5) group for the radial nerve and at the metacarpal site for the ulnar nerve, whereas the chronic pain (VAS < 5) group showed significantly higher thresholds only in the radial nerve. The differences were small. There were no significant differences in VPT for the median nerve. The VPT values were not normally distributed, and so we also performed a multivariate regression model analysis with logarithmically transformed vibration thresholds; the results were similar. For simplicity, we present only nonlogarithmic values.

**Table 2:** Multivariate regression analysis of vibrotactile perception thresholds

	Median nerve				Ulnar nerve				Radial nerve	
	Distal pad dig II		Palmar aspect of metacarpal I-II		Distal pad dig V		Metacarpal V		Metacarpal II	
	β	(95% CI)	β	(95% CI)	β	(95% CI)	β	(95% CI)	β	(95% CI)
**Model A**										
**Intercept**	0.204	(−0.109; 0.517)	0.279	(−0.158; 0.716)	0.213	(−0.305; 0.731)	**0.227**	**(0.036; 0.419)**	**0.274**	**(0.107; 0.441)**
**Chronic pain (all)**	−0.025	(−0.163; 0.113)	0.055	(−0.137; 0.248)	0.124	(−0.105; 0.352)	**0.091**	**(0.007; 0.175)**	**0.106**	**(0.032; 0.179)**
**Age**	**0.011**	**(0.004; 0.018)**	**0.016**	**(0.006; 0.026)**	**0.016**	**(0.003; 0.028)**	**0.006**	**(0.002; 0.011)**	**0.004**	**(0.001; 0.008)**
R^2^		0.089		0.107		0.090		0.148		0.152
**Prob > F**		**0.012**		**0.004**		**0.010**		**<0.001**		**<0.001**
**Model B**										
**Intercept**	0.202	(−0.247; 0.651)	0.363	(−0.265; 0.992)	0.042	(−0.702; 0.785)	0.220	(−0.055; 0.495)	**0.262**	**(0.023; 0.501)**
**Chronic pain (all)**	−0.015	(−0.156; 0.125)	0.059	(−0.139; 0.256)	0.110	(−0.123; 0.343)	**0.091**	**(0.004; 0.177)**	**0.112**	**(0.037; 0.187)**
**Age**	**0.011**	**(0.004; 0.018)**	**0.016**	**(0.006; 0.026)**	**0.016**	**(0.003; 0.028)**	**0.006**	**(0.002; 0.011)**	**0.004**	**(0.0005; 0.008)**
**Stress**	−0.040	(−0.132; 0.052)	−0.009	(−0.138; 0.120)	0.050	(−0.103; 0.203)	0.002	(−0.055; 0.059)	−0.027	(−0.076; 0.022)
**Energy**	0.020	(−0.079; 0.119)	−0.023	(−0.162; 0.116)	0.031	(−0.133; 0.196)	0.002	(−0.059; 0.062)	0.016	(−0.036; 0.069)
R^2^		0.096		0.108		0.096		0.148		0.165
**Prob > F**		**0.046**		**0.027**		**0.045**		**0.004**		**0.002**

Covariates, *P*-values, and 95% CI are given.

In model A, chronic pain and age were explanatory factors (VPT = β_0_ + β_1_ x chronic pain + β_2_ x age). In model B, chronic pain, age, stress, and energy were explanatory factors (VPT = β_0_ + β_1_ x chronic pain + β_2_ x age + β_3_ x stress + β_4_ x energy).

The changes in vibrotactile perception thresholds after 4 h of work were small, went in both directions, and did not differ significantly between the chronic pain (all) group and the controls. A paired *t*-test for individual VPT (all subjects) between the first and second test gave mean differences of −0.08μm (SE 0.03, *P* = 0.02) for the median nerve/distal pad, 0.01 μm (SE 0.03; *P* = 0.71) for the median nerve/metacarpal, 0.04 μm (SE 0.05; *P* = 0.40) for the ulnar nerve/distal pad, 0.01 μm (SE 0.01; *P* = 0.40) for the ulnar nerve/metacarpal, and −0.00 μm (SE 0.01; *P* = 0.99) for the radial nerve.

In the multivariate regression model, neither stress nor energy influenced the VPT; the group difference in VPT did not change when adjusted for stress and energy (Table 2). We also dichotomized the variables for stress and energy at the scale values which represent the neutral point of the respective scale, and there was still no influence on VPT or change in VPT difference between groups.

### Nerve Conduction Test

There was no significant difference in any parameter of the nerve conduction test between the chronic pain (all) group and the controls (Table 3). Specifically, there was no difference between the two groups in motor or sensory conduction velocity in the median nerve and no difference in sensory conduction velocity in the ulnar nerve. Furthermore, there was no significant difference in any parameter of the nerve conduction test between the controls and any pain group, and for simplicity we do not present the figures for the subgroups. No difference between the groups was seen after adjustment for age and body height. Temperature during the examination was similar in both the chronic pain (all) group (34.9 ± 1.0°C) and the control group (34.7 ± 1.1°C) (Table 3), and it remained stable during the whole measurement period.

**Table 3:** Nerve conduction test

	Chronic pain (all)		Controls			
	Mean	SD	Mean	SD	95% CI group difference	*t*-test *P*-value
Motor examination						
Median Nerve						
Velocity (m/s)	57.4	2.9	57.3	2.9	−1.3; 1.2	0.94
Amplitude (elbow) (mV)	10.1	2.4	9.6	2.9	−1.6; 0.6	0.39
Amplitude (wrist) /mV)	10.5	2.4	10.1	3.0	−1.5; 0.8	0.52
Distal latency (ms)	3.3	0.3	3.4	0.3	−0.1; 0.2	0.53
F-latency (ms)	21.7	1.2	21.7	1.2	−0.5; 0.5	0.98
Sensory examination						
Distal latency						
Palm-wrist, dig II (median nerve) (ms)	1.2	0.6	1.3	0.2	−0.1; 0.3	0.50
Palm-wrist, dig III (median nerve) (ms)	1.3	0.2	1.3	0.1	−0.1; 0.1	0.73
Finger-wrist, digV (ulnar nerve) (ms)	2.2	0.1	2.2	0.2	−0.1; 0.1	0.75
Velocity						
Finger-wrist, dig II (median nerve) (m/s)	51.3	4.4	51.8	3.3	−1.2; 2.3	0.51
Finger-wrist, dig III (median nerve) (m/s)	49.2	4.0	49.2	3.0	−1.6; 1.6	0.96
Finger-wrist, dig V (ulnar nerve) (m/s)	56.1	3.1	55.9	3.7	−1.6; 1.2	0.82
Suralis nerve(m/s)	52.3	4.6	51.1	4.8	−3.3; 0.9	0.25
Temperature						
Fingerpad, dig IV (°C) (during sensory examination dig III)	34.9	1.0	34.7	1.1	−0.6; 0.3	0.51

No significant difference in any parameter of the nerve conduction testing between chronic pain(all) group and controls.

## DISCUSSION

The main findings in this study were: (1) perceived stress and energy did not influence the VPT; (2) there was a small influence of chronic diffuse upper limb pain on VPTs, with increased thresholds seen in the area of the ulnar and radial nerve in female workers with normal nerve conduction; and (3) there was no difference in conduction velocity between the groups.

The findings from the mood adjective list revealed that the group with chronic diffuse upper limb pain and VAS ≥ 5 had significantly higher stress scores compared to the control group. There was no significant difference in energy between groups. Neither perceived stress nor perceived energy appeared to influence the VPT in our study groups. It is not clear whether perceived stress and energy influence the subject's attention, concentration, and/or motivation during vibration threshold testing. The vibration threshold test is a psychophysical test, and our study does not support the theory that the increased VPT in subjects with chronic pain is due to mood. To our knowledge, there has been no previous clinical study on the influence of mood in vibration threshold measurements.

The findings regarding VPT are partly in line with earlier studies that showed increased VPT within the areas of the ulnar and median nerves, but not the radial nerve, in patients with nonspecific arm pain.^1^–^3^ On the other hand, Johnston et al.^4^ found increased VPT not only within median and ulnar areas but also in the radial nerve area in office workers who experienced neck pain with and without arm pain, and Tucker et al.^7^ found elevated VPT within the areas of all three nerves in patients with diffuse upper limb pain disorder.

The mean differences in VPT between the chronic pain (VAS ≥ 5) group and the controls in our study were small: 0.61 versus 0.46 μm for the radial nerve and 0.64 versus 0.49 μm for the ulnar nerve/metacarpal. In the radial nerve area the numerical difference in VPT between the chronic pain (VAS ≥ 5) group and the control group was approximately the same as that reported by Johnston et al.^4^ and approximately one-third of that reported by Tucker et al.^7^ The corresponding difference for the metacarpal bone within the area of ulnar nerve was approximately the same as those reported by Greening et al.^1^,^2^ and Johnston et al.^4^ and approximately half of those reported by Jensen et al.^3^ and Tucker et al.^7^ The difference between our chronic pain (all) group and the controls was smaller than the difference between the chronic pain (VAS ≥ 5) group and the controls, and the difference was even smaller after adjustment for age in the multivariate linear regression model. Thus, in our study, chronic moderate and severe pain had a greater influence on VPTs than did chronic mild pain.

The minor discrepancies between the studies mentioned above and our study might be related to differences in upper extremity disorders. Since the participants in our study were still working (not on sick leave) and were not referred by other physicians, their ongoing pain may have been less intense or of different etiology. Another possibility would be differences in methodology. In order to increase accuracy and reproducibility in the vibration threshold testing, the examinations in our study were performed by a single trained assistant who was blinded both to the group of the subjects and to the results of the preceding examination. The subjects were seated comfortably and examined in a quiet room without distractions. It is unclear whether the conditions were similar in the aforementioned studies. There was a small decrease in VPT between the first and second test at the distal pad within the area of the median nerve. The index finger was the first finger to be measured in the vibration threshold test and we hypothesize that the decrease could be due to a learning effect.

Among a group of computer users with symptoms in the hand and forearm, Jensen et al.^3^ found indications of entrapment of median and ulnar nerves that could not be explained by a general increase in perception threshold, since no difference among groups was found at the recording site representing the radial nerve. In the present study, all nerve conduction velocities and hand temperatures were similar in the chronic pain (all) and the control groups, and therefore the increased VPTs in the chronic pain (all) group are unlikely to have been related to dysfunction or entrapment of large myelinated peripheral nerve fibers at the wrist.

However, one must bear in mind that only the fastest of the large myelinated fibers, and thus a limited proportion of the whole nerve fiber population, are examined in nerve conduction studies. Consequently, it is theoretically conceivable that a subgroup of myelinated fibers may be afflicted in spite of normal nerve conduction. Fiber types other than large myelinated fibers such as small myelinated and unmyelinated fibers may also be involved in chronic diffuse upper limb pain. The function of the smaller fibers that convey sensations of pain and temperature can be assessed by measurements of the psychophysical thresholds of those sensations.

Greening and Lynn^1^ suggested that increased VPT could be ascribed to computer work. However, in our study this possibility is not likely, as a previous study we conducted of the same participants revealed no difference in VPTs or nerve conduction between the secretaries, who used the computer intensively, and the nurses, who seldom used the computer.^11^

In office workers who experienced neck pain with and without arm pain, Johnston et al.^4^ found increased VPT within the areas of the median, ulnar, and radial nerves. This is compatible with peripheral nerve dysfunction at a proximal (e.g., brachial plexus) level and/or altered sensory processing due to central inhibition. Laursen et al.^6^ found raised vibration thresholds in the contralateral limb in patients with upper limb disorders, supporting the theory of central nervous alteration. Tucker et al.^7^ found global elevation of VPTs in subjects with carpal tunnel syndrome or upper limb disorders, and concluded that this is consistent with a physiological inhibitory mechanism, common to both conditions, which appears to be related to CNS perception of chronic pain rather than a specific peripheral pathology.

Based on the results of our study and those of the previous studies mentioned above, we find it reasonable to propose that pain may lead to a global increase of upper limb VPTs. This does not exclude the possibility that pain sometimes is due to a peripheral nerve affliction but rather that central and peripheral mechanisms may be involved at the same time and contribute (in varying proportions) to both the pain and the increase in VPTs. The gate control theory of pain suggests that concurrent tactile stimuli may decrease the perception of pain.^27^ However, Apkarian et al.^28^ have proposed the opposite; that is, that there is an inhibitory effect of nociceptive input on the perception of touch via a thalamic “touch gate.” The touch gate theory is supported by findings of decreased sensitivity to light touch in experimentally induced pain in healthy subjects in the area of referred pain.^29^ Moreover, improved sensitivity to light touch has been reported following relief of pain in patients with chronic pain.^30^

To our knowledge, little information is available regarding the optimal sites for determination of VPTs in the hand. The fingertips and the palm have higher receptor density than the dorsum of the hand.^31^–^33^ In our study we found increased VPT only at the dorsum of the 5th metacarpal bone and the dorsum of the 2nd metacarpal bone and not in the fingertips or the palm. We hypothesize that the reason for these results is that there is a delicate balance in the thalamic “touch gate” between the excitatory input from somatosensory receptors and inhibition from nociceptive stimuli. Because of the higher receptor density in the fingertips and the palm compared to the dorsum of the hand, the sensory input from fingertips and palm may be able to outbalance the inhibition from the pain, and therefore VPT is normal in these areas. In contrast, the sensory input from the dorsum of the hand is weaker, and therefore the VPT increases in this area.

### Limitations of the Study

The reason for the nonpositive finding of a relationship between mood and VPT may be due to a lack of variability in mood. However, the variability in perceived stress is of the same magnitude as described for female workers in a production system.^19^

Lack of power could be the reason why we found no significant difference in VPT between the chronic pain (all) group and the controls within the area of the median nerve.

The menstrual phase was not addressed. Whether physiological changes accompanying menstrual cycles can change sensory perception thresholds has been unclear; however, a study of menstrual phase and current perception thresholds did not see any changes across the menstrual cycle.^34^

In conclusion, chronic diffuse upper limb pain is associated with a small elevation of vibrotactile perception thresholds in the areas of the ulnar and radial nerves. Perceived stress and energy before the vibration threshold testing did not influence the thresholds. Although a peripheral mechanism cannot be excluded, our findings support the idea that increased vibration perception thresholds in chronic diffuse upper limb pain may be secondary to pain and be brought about by a central nervous mechanism.

## Abbreviations:

EMG: electromyography
SCV: sensory conduction velocity
VAS: visual analog scale
VPT: vibrotactile perception threshold
